# Precision of posttraumatic primary orbital reconstruction using individually bent titanium mesh with and without navigation: a retrospective study

**DOI:** 10.1186/1746-160X-9-18

**Published:** 2013-07-02

**Authors:** Harald Essig, Lars Dressel, Majeed Rana, Madiha Rana, Horst Kokemueller, Martin Ruecker, Nils-Claudius Gellrich

**Affiliations:** 1 Department of Craniomaxillofacial Surgery, Hannover Medical School, Carl-Neuberg-Str. 1, D-30625, Hannover, Germany

**Keywords:** Orbital fractures, Computer-assisted surgery, Orbital volume, Imaging analysis platform

## Abstract

**Background:**

The aim of orbital wall reconstruction is to reestablish anatomically exact orbital volumes to avoid long-term complications. Navigation could facilitate complex reconstructions.

**Methods:**

Quality of the orbital reconstruction (n = 94) was measured based on (A) volume changes and (B) on 3D shape deviations compared to the unaffected side. Volume analysis included segmentation of the orbital cavity in the pre- and post-operative 3D data set (VoXim®, IVS Solutions, Germany), and shape analysis was performed by vector-based 3D tools (Comparison®, 3Dshape, Germany).

**Results:**

Orbital volume of the unaffected side ranged from 26.6 ml ± 2.8 ml in male and 25.2 ml ± 2.6 ml in female (CT). Significant orbital enlargement was found in orbital fractures with involvement of the posterior third of the orbital floor and in comminuted fracture pattern. Reconstructed orbital volume ranged from 26.9 ± 2.7 ml in male and 24.26 ± 2.5 ml in female (CBCT). 3D Analysis of the color mapping showed minor deviations compared to the mirrored unaffected side.

**Conclusion:**

Measurements demonstrate that even in comminuted orbital fractures true-to-original reconstruction is feasible.

## Background

The aim of three-dimensional reconstruction of orbital walls is to reconstruct true-to-original and thus avoid long term complications. Among a broad scope of sequelae in orbital trauma, two typical major complications occur: enophthalmos and hypoglobus [1]. Both are the most common persistent complications of orbital trauma and are based on a posttraumatic enlarged orbital volume [2-5]. Inadequate reconstruction can restrict normal function and aesthetics of the midface [6]. The goal of the reconstruction procedure is therefore the reduction of the enlarged bony orbit. Computer-assisted pre-operative planning and intra-operative navi-gation are very effective tools for primary and secondary reconstruction of the orbit [7,8]. Based on the virtually mirrored bony orbit of the unaffected side the surgeon is able to control the reconstruction of the orbit intra-operatively using the navigation device [6,9]. Titanium mesh implants individually bent promise a filigree reconstruction even of the complex shape of orbital walls [10,11].

Computed tomography (CT) is considered to be the standard imaging in orbital trauma. Post-operative imaging is recommended to control orbital reconstruction [12]. Position of the radioopaque reconstruction material (titanium mesh) could also be visualized in Cone beam computed tomography (CBCT). This means lesser radiation compared to CT scan [13].

Assuming a facial symmetry, our aim was firstly, to examine to which exactitude a true-to-original reconstruction of orbital volume and shape is possible, secondly to assess how much accuracy can be achieved by intraoperative navigational control, and thirdly to determine the difference in values between two imaging techniques (CT and CBCT) and to compare the pre- and post-operative volumes of the unaffected, affected and reconstructed orbit in patients with primary reconstruction of orbital fractures using individually bent titanium mesh with and without navigation.

## Materials and methods

The patient population consisted of patients with unilateral orbital wall fractures who were operated at the Medical University Hospital in Hannover between January 2007 and July 2010. A further requirement for inclusion into the study was the availability of a valid pre-operative CT scan and a post-operative CBCT scan. Baseline data collected included age, gender, injury side, fracture type and location, and use of navigation device. The algorithm for intra-operative reconstruction implied preforming of the orbital floor mesh plate (3 mm, Synthes®, Paoli, USA) on either an artificial sterile skull (Synthes®, USA) or patient specific individual stereolithographic models. Depending on the extent of the reconstruction, a navigation system was used (VoXim®, IVS Solutions, Germany and Brainlab®, Feldkirchen, Germany). Our indications for navigation in primary orbital reconstruction are fractures of the medial orbital wall, orbital floor fractures of the posterior third, complex comminuted orbital fractures and fractures with involvement of the transition area between medial wall and orbital floor.

Indications for Navigation-assisted primary orbital reconstruction

(Hannover Medical School, Germany)

Fractures of the medial orbital wall

Fractures of the posterior third of the orbital floor

Complex comminuted orbital fractures

Orbital wall fractures including the transition zone between medial orbital wall and orbital floor

The operative technique of reconstruction was identical in the navigation group (Navi) in the conventional (conv). The individually preformed titanium orbital floor mesh plates were inserted using a retroseptal transconjunctival approach and fixed with 1.0 or 1.3 titanium microscrews (Synthes®, USA).

The orbital volume was measured pre-operatively on the unaffected and affected orbit by means of CT and post-operatively by means of CBCT using an imaging analysis platform (VoXim®, IVS Solutions AG, Chemnitz, Germany). The pre-operative CT scan was obtained with the following minimum requirement: slice thickness of maximal 1 mm. The parameters for the post-operative CBCT (NewTom DVT 9000, NewTom Deutschland AG, Marburg, Germany and OrangeDental PaxZenith 3D, Biberach, Germany) also include the minimum requirement of maximal 1 mm slice thickness (typically 0.3 mm).

**Figure 1:**
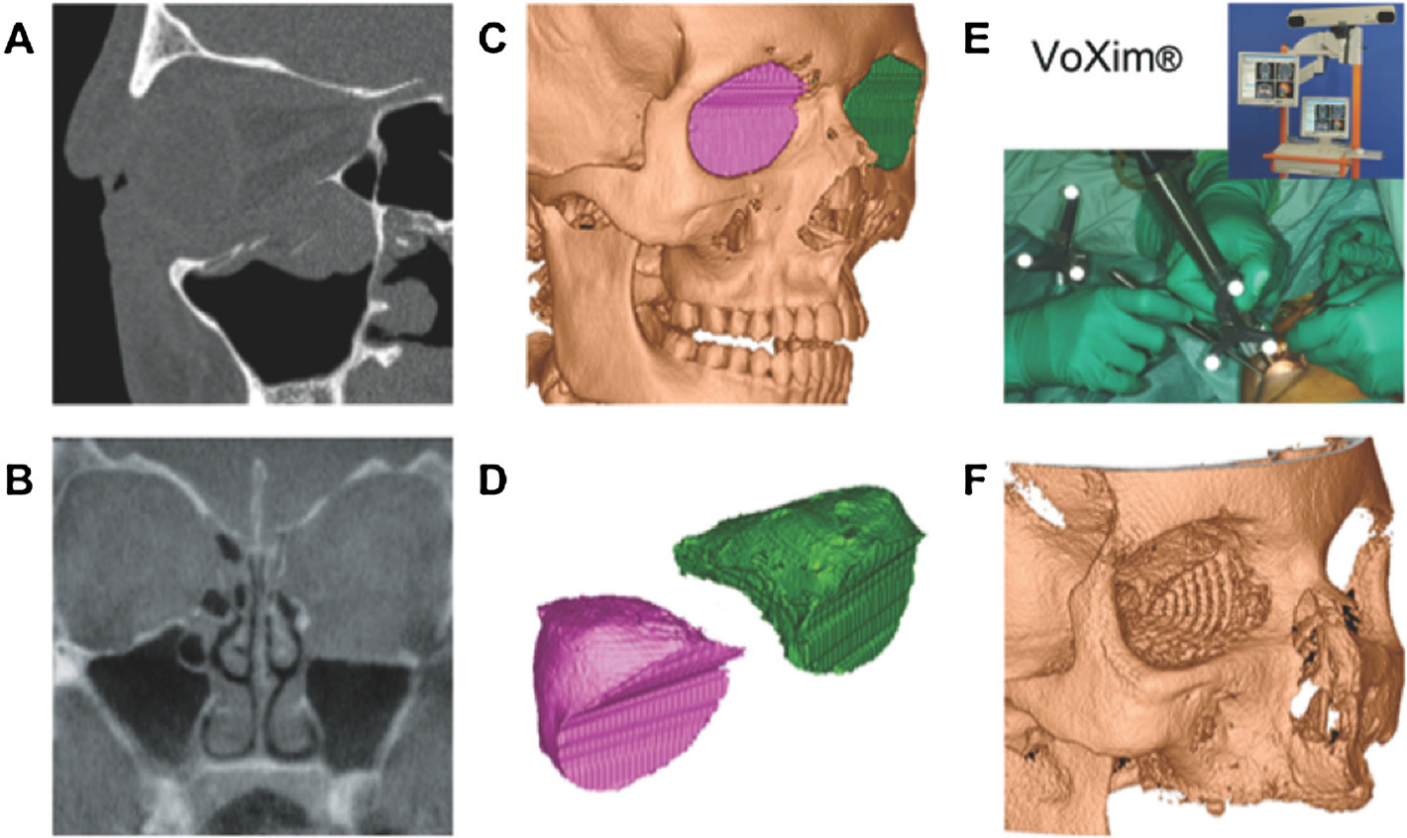
Image analysis in sagittal view (A) and coronal view (B), (C) pre-operative segmentation (CT scan), (D) both virtual segments, (E) intra-operative Navigation-assisted surgery using VoXim®, (F) post-operative control (CBCT-scan).

The volume of the orbital cavity was compared by segmentation based on the 3D data sets. Therefore we transferred DICOM-format CT and CBCT data sets to the software VoXim®. This software product allows for assessment of the patient’s individual anatomy in multiplanar and three-dimensional views (Figure 1). In three-dimensional reconstructions, volume data can be visualized by threshold value segmentation which figures objects with voxel values of a defined range and allows the measurement of defined subvolumes, by defining up to 8 segments and their independent movement.

**Figure 2:**
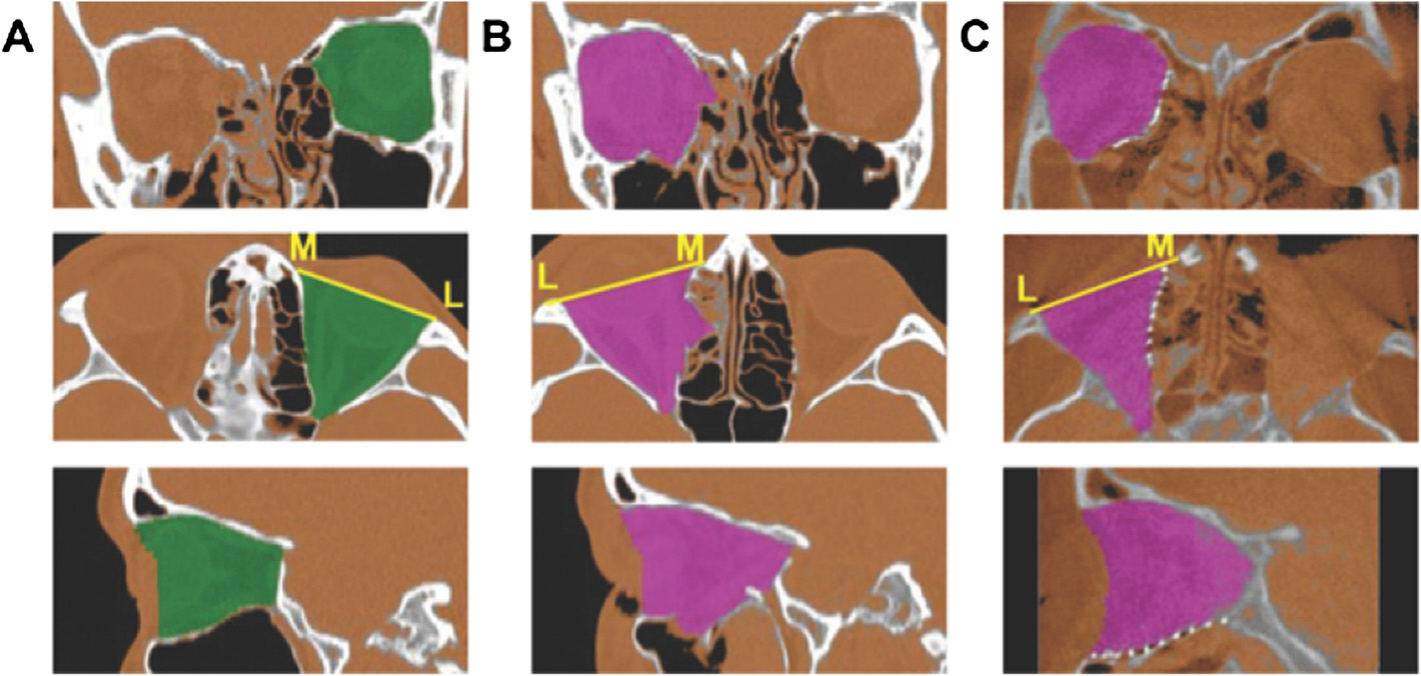
Segmentation of (A) unaffected, (B) affected, and (C) reconstructed orbit.

The bony orbit was electronically marked (segmentation) in axial slices and controlled in coronal and sagittal view for volume analysis. The anterior border of the orbit was defined by a straight line through the points L (lateral orbital rim) and M (medial orbital rim). For every axial slice, a subvolume can be assessed by the number of pixels within the defined region, each expressing a voxel value. The overall volume of the orbit is given in cm [3] (Figure 2).

**Figure 3:**
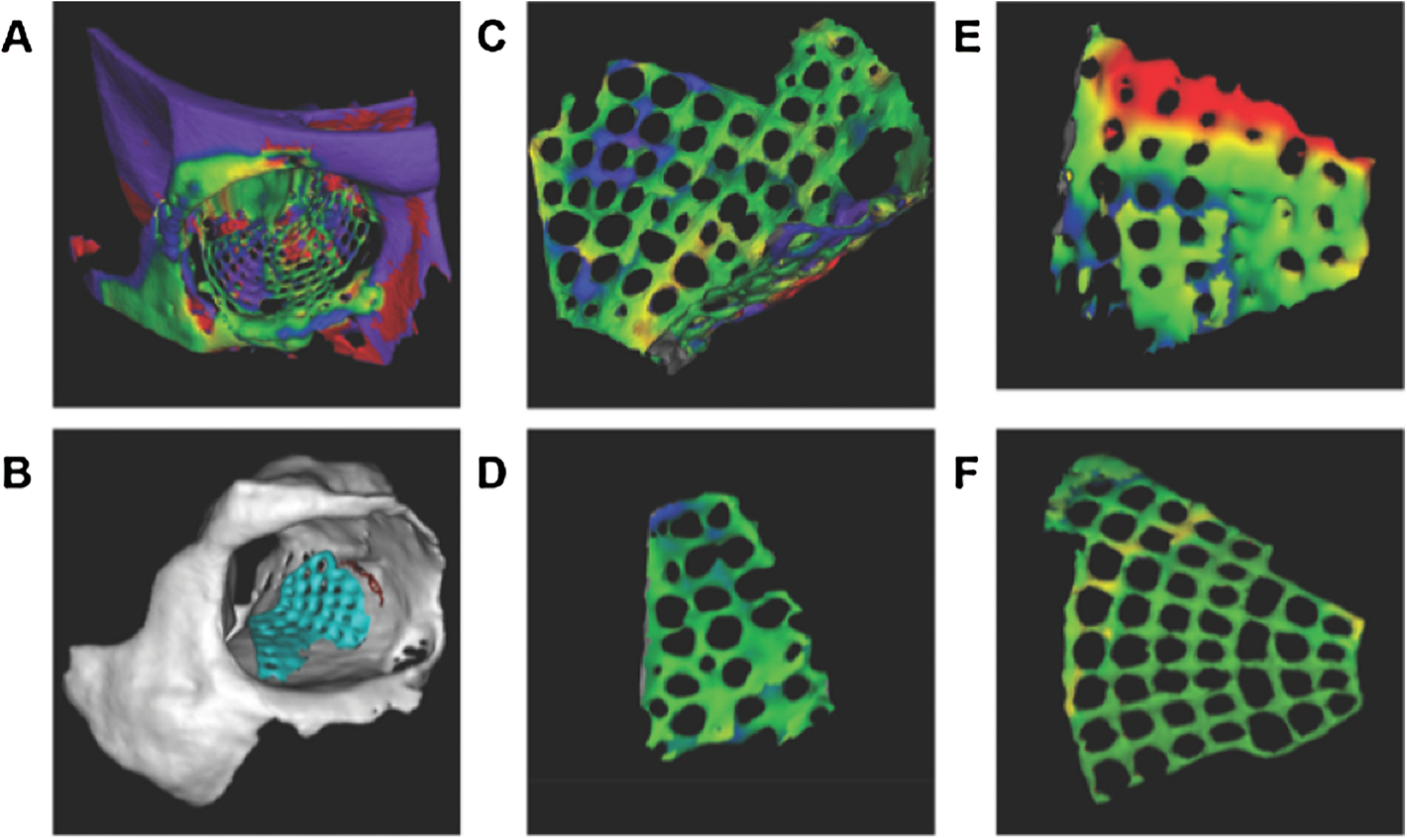
**3D shape analysis. (A)** Manually superimposed pre- and post-operative virtual models – before matching procedure. **(B)** after matching procedure. **(C)** typical segmentation of a titanium mesh implant. **(D)** medial orbital wall as area of interest (green signifies no differences compared to the virtual planning). **(E)** orbital floor with transition zone to the medial wall (in red; differences are up to 1.5 mm). **(F)** complete titanium mesh implant shows an excellent result.

Three-dimensional analysis presupposed two proper aligned virtual stereolithographic models (STL-data) of firstly the template (mirrored unaffected side) and secondly the post-operative result (reconstructed side). The template of the unaffected side was segmented with VoXim® (IVS Solutions, Germany) and mirrored to the affected side (group Navi; n = 60). This procedure is a well-established step of the Computer-assisted pre-operative planning (CAPP). The form of the template included the complete bony orbit with extent to the orbital roof. The reconstructed side was accordingly segmented and both files were saved in STL-format. The aligning was performed with Comparison® (3Dshape, Germany). The region of interest (ROI) for the superimposing was limited to the orbital roof and non-affected areas of the bony orbit. This restriction prevented the matching algorithm to include the reconstructed area. The output of Comparison® was a color-coded template (Figure 3). This template was split into the anatomical regions (medial orbital wall, orbital floor, lateral orbital wall) and respectively subdivided into anterior, central, and posterior third. The spectrum was evaluated with analySIS 1.0 (Soft Imaging Systems, Muenster, Germany) and volume and shape data analyzed with SPSS 18 (IBM, USA).

## Results

94 patients in the age of 18 to 84 years (38 ± 19.01 years) with unilateral orbital wall fractures were included. There were 58 (63.8%) men and 36 (36.2%) women. 52 (55.3%) fractures were located on the right side. Main causes of orbital trauma in the patient population were assaults (38%), road traffic accidents (25%), falls (22%), sports related injuries (6%), and non specified trauma (9%).

Fracture types include fractures of the anterior and central part of the orbital floor (n = 34), complete orbital floor fractures with involvement of the posterior ledge (n = 20) and complex fractures (n = 36) with involvement of the medial orbital wall respectively with the lateral orbital wall (n = 4). Following the authors’ indications, navigation was used in 60 patients (Navi), while 34 patients were treated without the help of navigation devices (conventional).

**Figure 4:**
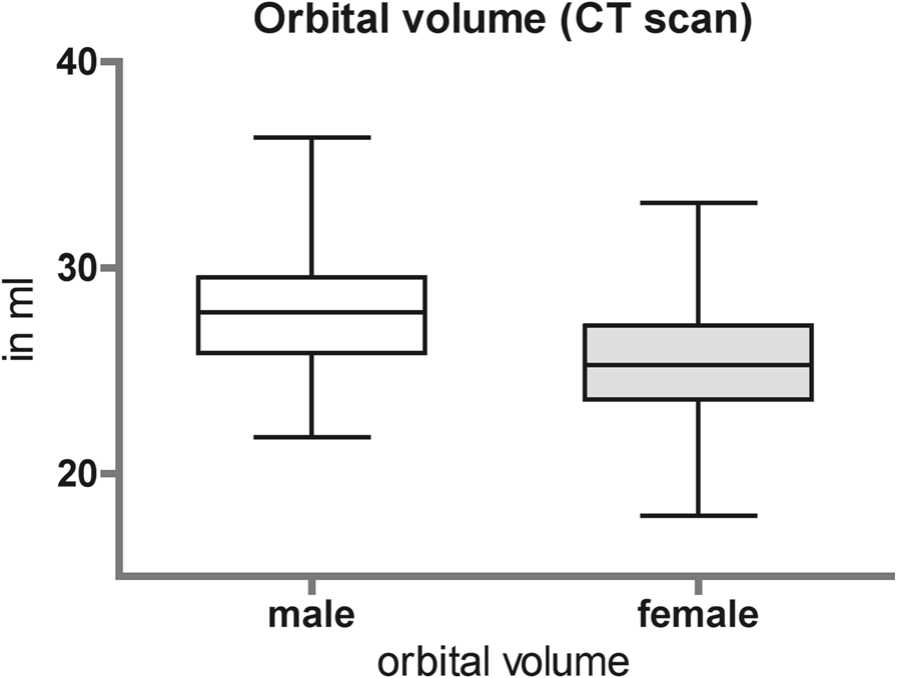
Orbital volume in adults (gender dependent).

**Figure 5:**
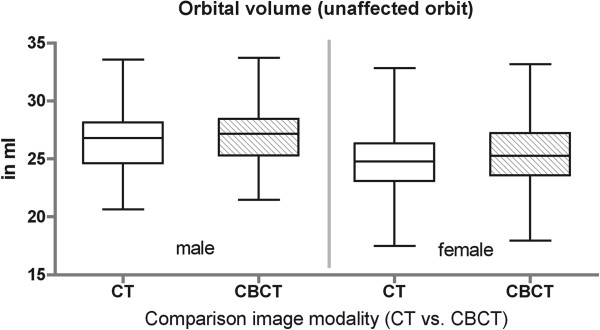
Comparison imaging modality (CT versus CBCT).

**Figure 6:**
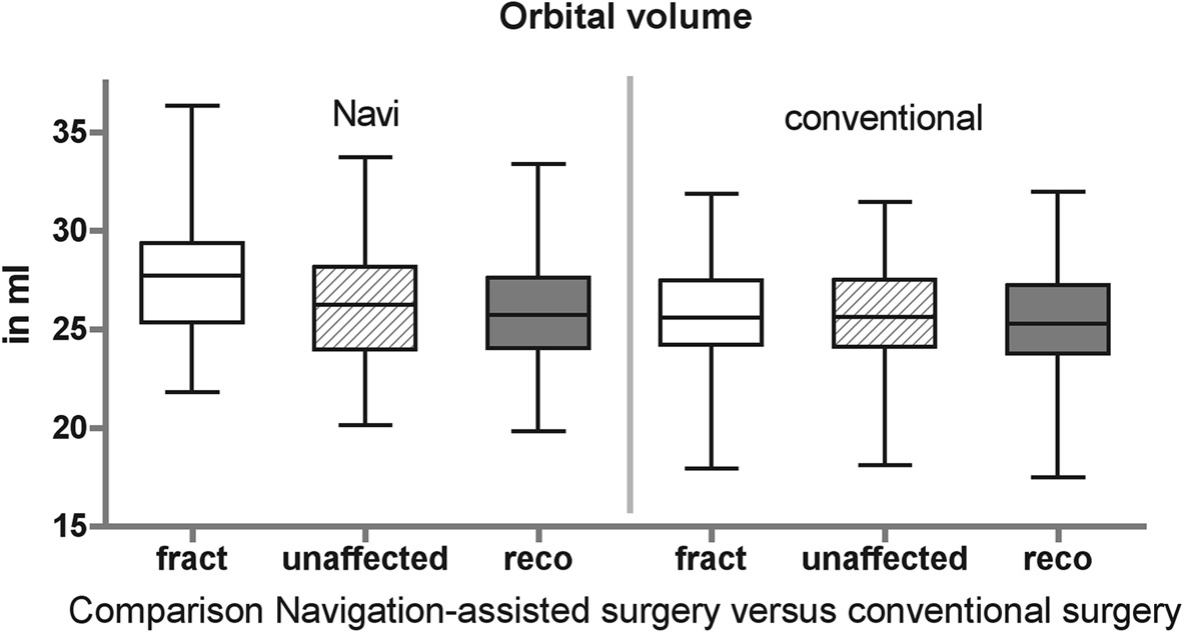
Comparison of Navigation-assisted surgery versus conventional surgery (fract) fractured orbit, CT, (unaffected) unaffected orbit, CBCT, (reco) reconstructed orbit, CBCT.

Volume of the unaffected orbit ranged from 26.6 ± 2.8 ml in male to 25.2 ± 2.6 ml in female (CT data set) (Figure 4). The post-operative imaging (CBCT) of the unaffected orbit was also measured and showed an orbital volume range from 27.4 ± 2.6 ml in male to 25.8 ± 2.6 ml in female (Figure 5). There is no statistical significance between CT and CBCT measurements and therefore comparison between pre- and post-operative data could be done. Volume of the affected orbit is measurably enlarged only in complex fracture pattern like in orbital floor fractures with involvement of the medial orbital wall (maximal 1.9 ml in male and 3.1 ml in female) or in orbital wall fractures including the posterior ledge (up to 3.4 ml in male and 3.2 ml in female) (Figure 6). The reconstructed orbital volume ranged from 26.9 ± 2.7 ml in male and 24.26 ± 2.5 ml in female (CBCT). If these results are broken down into the different groups, significant reduction could be achieved in complex orbital wall fractures (group Navi; 27.7 ± 3.4 ml to 25.7 ± 3.0 ml, p < 0.05) and non significant reduction in Group conv (25.6 ± 3.3 ml to 25.3 ± 3.3 ml).

**Figure 7:**
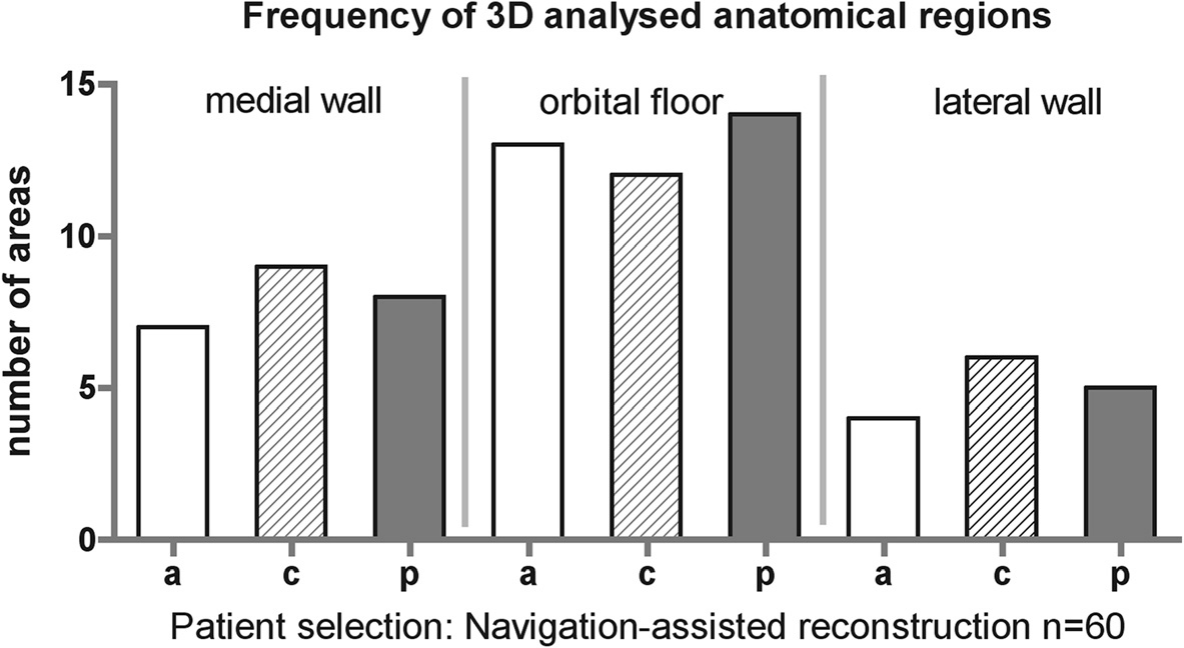
Analyzed anatomical regions (group Navi) (a) anterior third, (c) central third, (p) posterior third of the orbital wall.

**Figure 8:**
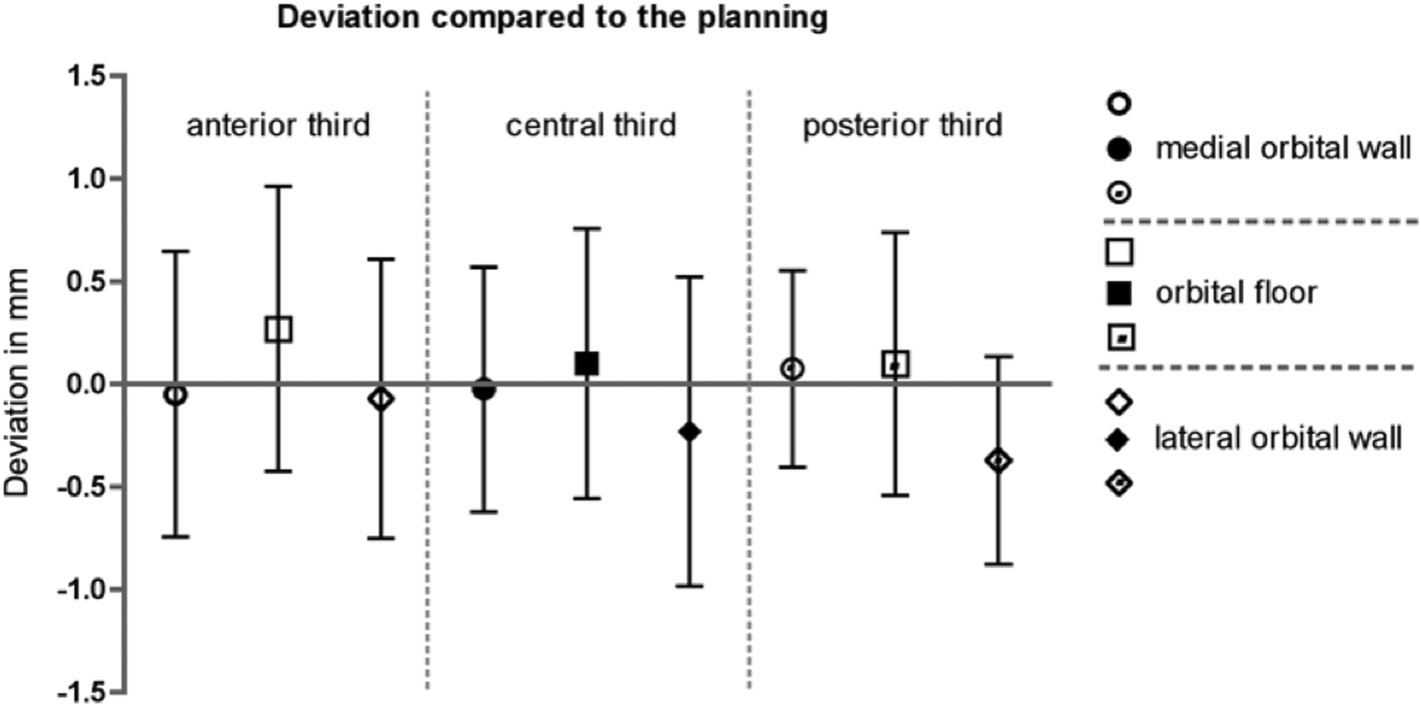
Deviation of reconstruction.

Three-dimensional analysis of the reconstructed area compared to the pre-operative planning is divided into 9 different regions: every orbital wall except of the orbital roof (medial orbital wall, orbital floor, lateral orbital wall) was sectioned into 3 proportional regions and labeled into anterior third, central third, and posterior third. Virtual plannings of the patients of the navigation group (Navi; n = 60) were superimposed to the post-operative result. Deviation of the minimal perpendicular distance was measured color-coded and converted into millimeters. The proportion of the reconstruction material (titanium mesh implant) compared to the size of the corresponding region was evaluated. Frequency of 3D analysed anatomical regions is displayed in Figure 7. Differences of the reconstructed medial orbital wall compared to the virtual planning were maximal in the anterior third (−0.05 ± 0.7 mm), of the orbital floor in the anterior third (0.27 ± 0.7 mm), and of the lateral orbital wall in the central third (−0.23 ± 0.753) (Figure 8).

## Discussion

The re-establishing of exact orbital volumes is a main goal in orbital reconstruction to avoid long-term sequelae [14-16]. Measurement of the orbital volume is reliable and three-dimensional shape analysis allows for post-operative reconstruction control as far as radioopaque reconstruction material was applied.

Orbital volume was significantly enlarged in complex orbital trauma that meets the authors’ indication for the use of navigation-assisted surgery. In minor orbital trauma, such as orbital floor fractures limited to the anterior third, the degree of the orbital enlargement was not significant compared to the unaffected side. That could be because of the normal existing facial asymmetry [17] or bony variability [18]. Different studies with comparable volumetric methods state that enophthalmos is mainly related to enlargement of the bony orbit and exact reconstruction and repositioning of orbital soft tissue will correct or at least significantly improve post-traumatic enophthalmos, assuming that the volume of orbital soft tissue remains constant following trauma [19-21]. Post-traumatic or post-operative fat atrophy is also discussed to be partially responsible for post-traumatic or post-operative enophthalmos [22,23]. Statements of linear relation between orbital volume increase and sagittal projection of the eye ball might therefore be critically evaluated [2,6,24].

According to the literature, there are different methods for the measurement of orbital volumes [25]. The difficulty is the definition of the anterior border of the bony orbit [26]. For comparability reasons the definition of the anterior border is identical to Bite and Schuhknecht [22,27]. Schuhknecht et al. reported a mean volume of 26.8 ml in 11 patients [22]. In our patient selection there was a significant gender dependent difference in the orbital volume.

After surgery, either in minor trauma without or in complex trauma with the use of navigation, reconstructed orbital volume was measured and compared to the unaffected side assuming that the volume of the affected orbit did not differ prior to the trauma. Post-operative orbital volume of the reconstructed orbit was in this study identically equal to the unaffected side. This meets the criteria for true-to-original reconstruction.

Keeping in mind, that different geometrical forms may possess the same volume, three-dimensional analysis of the reconstructed orbit could provide information about the quality of reconstruction. Reconstructed medial and lateral orbital walls as well as orbital floors show only negligible deviations compared to the virtual planning. In-depth analysis of the different regions within the mentioned orbital walls present minimal deviations in the anterior third of the medial wall, the anterior third of the orbital floor, and in the central third of the lateral wall. These deviations could be based on the complex three-dimensional form of the nasolacrimal fossa and the concavity of the anterior orbital floor. The well defined posterior ledge supports titanium mesh position in the posterior third of the orbital floor.

Concluding, the true-to-original reconstruction in primary orbital trauma with titanium mesh implants appears to be satisfying with regard to volume re-establishment and adequate orbital shape. Post-operative 3D-imaging is necessary to improve surgical skills and to document post-operative results. An intra- or post-operative cone beam CT scan (CBCT) is sufficient to assess implant form and position with reduced radiation compared to conventional computed tomography.

## Conclusion

Regarding the results of the measurements, it could be demonstrated that even in comminuted orbital fractures true-to-original reconstruction is feasible.

### Consent statement

Written informed consent was obtained from the patient for publication of this case report and accompanying images. A copy of the written consent is available for review by the Editor-in-Chief of this journal.

## Abbreviations

3D: Three-dimensional; CT: Computed tomography; CBCT: Cone beam computed tomography; Conv: Conventional group; Navi: Navigation group; DICOM: Digital Imaging and Communication in Medicine; CAPP: Computer-assisted pre-operative planning; STL: Stereolithographic file format.

## Competing interests

The authors declare that they have no competing interest.

## Authors’ contributions

HE, LD, MR, MAR, HK, MRU and NCG conceived of the study and participated in its design and coordination. HE and LD made substantial contributions to conception and design of the manuscript as well as data acquisition. HE, MR, MAR, NCG have been involved in drafting the manuscript. NCG was involved in revising the manuscript. All authors read and approved the final manuscript.
